# Development of a high-field MR-guided HIFU setup for thermal and mechanical ablation methods in small animals

**DOI:** 10.1186/s40349-015-0035-6

**Published:** 2015-08-13

**Authors:** Martijn Hoogenboom, Martinus J. van Amerongen, Dylan C. Eikelenboom, Melissa Wassink, Martijn H. den Brok, Christina Hulsbergen-van de Kaa, Erik Dumont, Gosse J. Adema, Arend Heerschap, Jurgen J. Fütterer

**Affiliations:** Department of Radiology and Nuclear Medicine, Radboud University Medical Center, Geert Grooteplein-Zuid 10, 6500 HB Nijmegen, The Netherlands; Department of Tumor Immunology, Radboud University Medical Center, Nijmegen, The Netherlands; Department of Pathology, Radboud University Medical Center, Nijmegen, The Netherlands; MIRA Institute for Biomedical Technology and Technical Medicine, University of Twente, Enschede, Netherlands; Image Guided Therapy, Pessac, Bordeaux, France

## Abstract

**Background:**

Thermal and mechanical high intensity focused ultrasound (HIFU) ablation techniques are in development for non-invasive treatment of cancer. However, knowledge of in vivo histopathologic and immunologic reactions after HIFU ablation is still limited. This study aims to create a setup for evaluation of different HIFU ablation methods in mouse tumors using high-field magnetic resonance (MR) guidance. An optimized MR-guided-HIFU setup could be used to increase knowledge of the different pathologic and immunologic reactions to different HIFU ablation methods.

**Methods:**

Three different HIFU treatment strategies were applied in mouse melanomas (B16): a thermal (continuous wave), a mechanical (5 ms pulsed wave), and an intermediate setting (20 ms pulsed wave) for HIFU ablation, all under MR guidance using a 7 tesla animal MR system. Histopathologic evaluation was performed 3 days after treatment.

**Results:**

The focus of the ultrasound transducer could accurately be positioned within the tumor under MR image guidance, without substantial damage to the surrounding tissue and skin. All mice retained complete use of the treated leg after treatment. Temperatures of >60, <50, and <44 °C were reached during thermal, intermediate, and mechanical HIFU ablation, respectively. Thermal-treated tumors showed large regions of coagulative necrosis. Tumors of both the mechanical and intermediate groups showed fractionated tissue with islands of necrosis and some pseudocysts with hemorrhage.

**Conclusion:**

A stable small animal MR-guided HIFU setup was designed and evaluated for follow-up MR imaging and histopathologic responses of the treated tumors. This will facilitate further studies with a larger number of mice for detailed evaluation of the pathologic and immunologic response to different HIFU strategies.

## Introduction

During the last decades, new treatment options have been developed to treat localized tumors while sparing surrounding tissue. High intensity focused ultrasound (HIFU) is a non-invasive ablation technique to deliver large amounts of energy into a small millimeter-sized ablation zone, resulting in tissue destruction through cellular disruption and irreversible coagulation necrosis [1–3]. If correctly targeted, the energy of the ultrasound (US) beam penetrates the body without damaging the surrounding tissue. Using multiple focal ablations, different tumor volumes can be treated.

Both thermal and mechanical effects can be generated using HIFU [4]. The thermal effect is the consequence of absorption of ultrasound energy by the tissue and conversion into heat, inducing irreversible damage and local coagulative necrosis [4, 5]. The mechanical effect is created by the mechanical force and shock wave effect of the ultrasound wave which could result in acoustic cavitations [6]. With a low duty cycle, the local temperature rise remains limited due to thermal diffusion. Expansion and rarefaction of tissue due to high acoustic waves of a sufficient pressure magnitude result in alternating compression and expansion of the tissue. Subsequently, gas is extracted from the tissue and (micro)bubbles are created. The interaction of these bubbles with the ultrasound waves will result in disruption of the vascular structure [7, 8], connective tissue [9], and cellular damage. Extreme high ultrasound pressure can destroy the tissue in submicron fragments, so-called histotripsy [10–12] or boiling histotripsy (also known as millisecond boiling) [13–16].

While thermal HIFU ablation is already clinically used for more than a decade, mechanical HIFU ablation techniques are still in an early stage of development [6, 17]. Whether thermal or mechanical HIFU is the best possible way to treat tumors is not known. Moreover, the different pathological and immunological effects due to in vivo thermal or mechanical HIFU treatment are poorly understood. Immune effects have been observed as a response to the HIFU treatment, but further studies are needed to distinguish wound healing from relevant anti-tumor responses [9, 17, 18]. For evaluation of different tumor ablation techniques and their histopathologic changes and immunologic reactions, an adequate small animal HIFU system is required [19, 20], which can be used to test in vivo HIFU treatments [21].

HIFU guided by US or magnetic resonance imaging (MRI) is widely used [22]. The latter technique offers better anatomical information compared to US-guided techniques, better tumor detection, and a broader field of view to visualize both the treatment area and surrounding tissue [1]. Different MRI methods allow accurate targeting before treatment, real time temperature monitoring, and control of the energy deposited during treatment [1, 2, 21, 23, 24].

The purpose of this feasibility study was to create a controllable setup for evaluation and differentiation between different magnetic resonance (MR)-guided HIFU ablation methods in vivo. Three different methods are tested: (1) thermal (T-HIFU), (2) mechanical (M-HIFU), and (3) a mixed ablation method, termed intermediate temperature-HIFU (inT-HIFU) that resulted in a limited but significant temperature rise in between both other HIFU settings. Tumor response after the different ablation techniques is evaluated with T2-weighted (T2W) MR imaging immediately after treatment. Histopathologic evaluation is performed 3 days after treatment.

## Materials and methods

### Animal preparation

Nine- to eleven-week-old female C57Bl/6n wild type mice were purchased from Charles River Wiga (Sulzfeld, Germany) and kept under specific pathogen-free conditions in the Central Animal Laboratory of the Radboud University (Nijmegen, the Netherlands). All animal experiments were performed according to the guidelines and by approval of the Nijmegen Animal Experiments Committee.

Twenty-one mice were injected subcutaneously in the right leg with 0.5 × 10^6^ cells of the ovalbumin (OVA)-transfected murine melanoma cell line (B16F10-OVA) as previously described [19]. The volume of the tumors was calculated with the formula (A × B^2^) × 0.4 in which A is the largest and B is the shortest dimension, measured using a caliper. Eight to nine days after injection, a tumor diameter of more than 7 mm is reached and the mouse was ready for HIFU treatment.

Isoflurane gas at 3.5 % was used for anesthetic induction. The anesthetic concentration was adjusted to 1–2 % during the experiment in order to maintain the breathing frequency at 40–60 per minute. The body temperature was measured during the treatment using a rectal thermometer and maintained using a heated air flow device. The mice were euthanized 3 days after treatment by cervical dislocation.

### MR-HIFU treatment

#### Animal setup and protocol

An MR compatible animal HIFU system (Image Guided Therapy—IGT, Pessac, France) was used in all experiments. A 16-channel annular array HIFU transducer (3 MHz central frequency, 37 W acoustic peak power, 52 W electrical peak power, 86.58° aperture, 48 mm diameter, adjustable focus depth 30–80 mm) was embedded in a positioning system (MR compatible piezoelectric motors, 30 × 30 mm trajectory execution range, active transducer cooling system) which was controlled by trajectory planner software (Thermoguide, IGT, Pessac, France). The spherical cap of the transducer is divided into 16 concentric annuli of identical surface. Due to the cylindrical symmetry, the steering of the ultrasound focus is possible along the axis of the symmetry i.e., longitudinal positioning of the focal zone. Lateral positioning of the focal zone is processed due to transducer displacement via piezoelectric motors. The trajectory of ablation was manually implemented based on MR imaging.

The pressure field of the HIFU transducer was scanned using a 0.075 mm needle-type hydrophone (HPM075/1, Precision Acoustics, Dorchester, UK) and 50 μs pulses with an acoustical output power of 0.73 W (electrical output power of 1.04 W). The measurements were performed in a degassed water tank. The hydrophone was positioned into a 3D mechanical positioning system enabling a 0.05 mm measurement resolution.

**Fig. 1:**
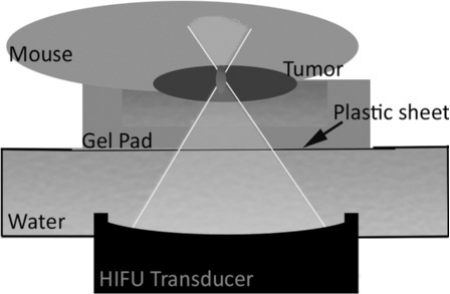
An schematic overview of the high intensity focused ultrasound (HIFU) setup

For good acoustic coupling of the US beam, the tumor area was shaved and the remaining hair was removed using standard hair removal cream. The mouse was positioned on top of an in-house made gel pad in lateral decubitus position, with the tumor inside a cavity (approximately 3.5 × 3.5 × 1 cm) made in the gel pad, and filled with degassed water. The gel pad was positioned on top of a plastic sheet covering the HIFU transducer. The space between the plastic sheet and the transducer was filled with degassed water for good acoustic coupling (Fig. 1).

**Fig. 2:**
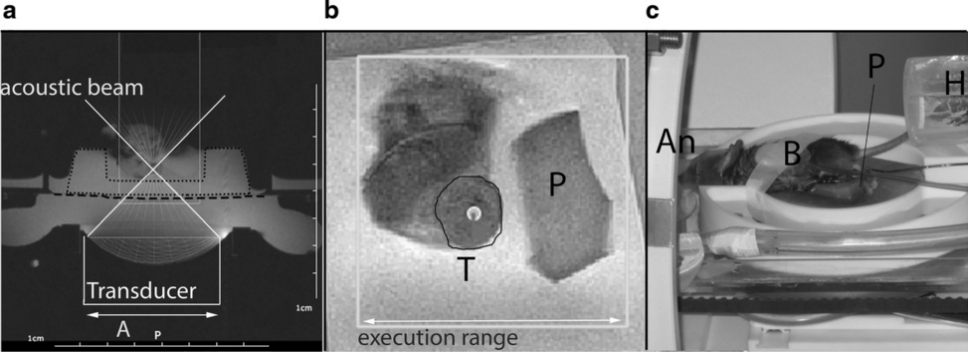
Images of the setup and treatment planning. **a** T1 weighted magnetic resonance (MR) localizer (repetition time/echo time = 196/4 ms) for visualization of possible air bubbles within the ultrasound beam. The *white line* is the acoustic beam overlay. *Dashed line* is the plastic sheet on top of a water bath. *Dotted line* indicates the gel pad. Focal length ranges between 30–80 mm, acoustic window 48 mm in diameter. **b** T2 weighted MR images (repetition time/echo time = 3350/26 ms) to determine the tumor (T) and focus spots (dot within the tumor) using the trajectory planner software (Thermoguide, Image Guided Therapy, Pessac, France). The *white box* indicates the execution range (30 × 30 mm) of the high intensity focused ultrasound system. The mouse leg with tumor is visualized in a water bath. A small phantom (P) is positioned next to it for focus corrections. **c** Mouse positioned on its side, with the tumor inside a water bath and the nose of the mouse within a tube for anesthesia (An). The phantom cube (P) is position next to the mouse. Breathing rate (B) and rectal temperature are measured during treatment; an air-heater (H) is connected to the rectal thermometer to maintain the mouse core temperature

The HIFU system was calibrated with the MRI in each experiment for localization of the focus of the US beam. A small phantom (approximately 1 × 1 × 1 cm) was positioned next to the tumor, within the cavity of the gel pad (Figs. 1 and 2). Preceding the HIFU treatment of the tumor, a test HIFU pulse (4 s, 28 W acoustic output power) was sent to this phantom cube. With MR thermometry, the focus region was visualized and compared to the preset coordinates (Fig. 2b). Any possible mismatch was corrected for.

To visualize the border between treated and non-treated tumor tissue, three to six focused spots were positioned in the center of the tumor depending on tumor size and treatment method used. Thermal diffusion, which occurs in thermal HIFU treatment, results in an increased area of tumor destruction of each ablation compared to the M-HIFU or inT-HIFU treatment. Therefore, three to four focal sonications, 2 mm apart, were used during thermal treatment (T-HIFU) and a maximum of six focal sonications (1.5 mm apart) for M-HIFU and inT-HIFU treatment. Six mice were subjected to T-HIFU with a continuous wave HIFU pulse of 4 s per sonication. Five mice were treated with the inT-HIFU method, 120 pulses of 20 ms (inT-HIFU treatment) with a pulse repetition frequency of 4 Hz. Six mice received a more mechanical HIFU ablation method (M-HIFU) with 500 pulses of 5 ms with a pulse repetition frequency of 4 Hz. For all methods, T-HIFU, M-HIFU, and inT-HIFU, an acoustic output power of 40–48 W was used, which correlates approximately to an acoustic dose per sonication of 160, 100, and 96 J. One mouse was excluded due to bursting of the tumor before treatment was feasible. Three tumor-bearing mice did not receive any treatment and were used as a control group.

The HIFU settings were directed by the temperature rise during ablation and the corresponding response in phantoms and ex vivo experiments. The T-HIFU group received a continuous US wave causing the temperature to rise within the focal region. Ex vivo experiments showed, with these settings, an increase of a minimum of 30 °C. The pulsed waved character of M-HIFU and inT-HIFU methods was used to limit the temperature rise and give a more mechanical character to the treatment method. The same pulse repetition frequency (4 Hz), but a different pulse duration and amount of pulses, was chosen for the M-HIFU and inT-HIFU methods (500 pulses of 5 ms and 120 pulses of 20 ms, respectively). The HIFU on time was similar, i.e., 2400 and 2500 ms, and the temperature increased in ex vivo specimens was 8 and 15 °C.

#### MR imaging and thermometry

For MR imaging and treatment guidance, a 7 tesla horizontal wide bore (20 cm) animal MR scanner was used (ClinScan, Bruker Biospin GmbH, Rheinstetten, Germany), with an additional body coil insert (free bore diameter 15 cm). Before treatment, an axial susceptibility sensitive T1 weighted (T1W) gradient echo (GRE) sequence (repetition time/echo time (TR/TE) of 196/4 ms, flip angle (FA) of 25^0^, voxel size 0.51 × 0.51 × 2.50 mm) was acquired to check for the presence of air bubbles within the line of the acoustic beam (Fig. 2a). Mice were repositioned if gas bubbles were present. Subsequently, localized T1W MR images were acquired with a gradient echo fast low angle shot (GRE-FLASH) sequence (TR/TE = 236/4 ms, FA = 25^0^, voxel size 0.78 × 0.78 × 1.50 mm) and transferred to the HIFU workstation for synchronization of the coordinates of the HIFU transducer with the coordinates of the MR scanner. Before and after the HIFU treatment, anatomical T2W MR images were acquired for tumor localization (Fig. 2b), trajectory planning, and tumor response analysis, using a turbo spin echo (TSE) scan (TR/TE = 3350/26 ms, FA = 180^0^, voxel size 0.25 × 0.25 × 1 mm, TSE-factor 7). The T2W images were sent to the HIFU trajectory planning software (Thermoguide, IGT).

**Fig. 3:**
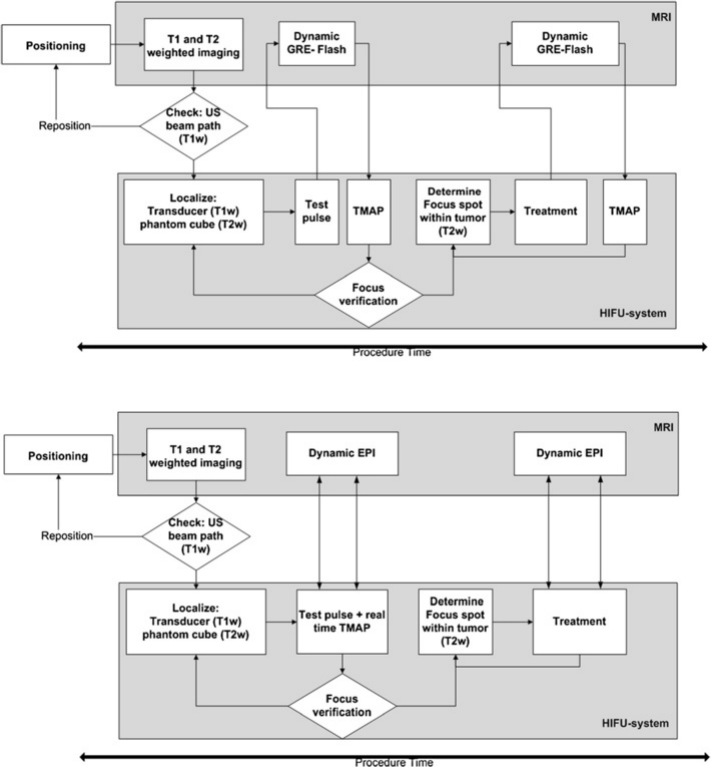
Workflow chart of the high intensity focused ultrasound treatment using both magnetic resonance thermometry methods with the gradient echo flash (GRE-FLASH) and echo planner imaging (EPI) sequence. GRE-FLASH uses a method with interleaved ablation and temperature calculations. With EPI, the temperature is defined during the ablation. TMAP: temperature map, T1W/ T2W: T1/ T2 weighted images

The ablation process was guided by MR thermometry. Two different thermometry sequences have been tested. In three mice of each group, an interleaved GRE-FLASH sequence (TR/TE = 40/4 ms, FA = 25^0^, voxel size 0.78 × 0.78 × 1.5 mm^3^, matrix size 128 × 128, five slices with distance factor 20 %, temporal resolution 3.8 s) was used. Data was manually transmitted to the HIFU system, and temperature maps were calculated and visualized after each ablation instead of during the ablation (Fig. 3). Cooling time of at least 30 s before the next sonication was stated, and before each focused sonication a new reference scan was made. For the other mice (three per group) an echo planar imaging (EPI) sequence (Siemens, Erlangen, Germany) was used (TR/TE = 30/4.5 ms, FA = 15^0^, voxel size 1 × 1 × 1.5 mm^3^, 3 axial, and 1 coronal slices, 20 % inter-slice distance, temporal resolution 1.9 s). Temperature maps were automatically sent to the HIFU system and immediately visualized (Fig. 3), also for this sequence, at least 30 s cooling time after each sonication was included. A proton resonance frequency shift method was used to determine temperature changes [23, 25–27]. The temperature in the treated area was determined by adding up the calculated temperature rise to the body temperature, measured by a rectal thermometer. The thermal dose map was calculated using the Sapareto and Dewey model [5] and visualized with a threshold defined at the lethal dose of the tissue in 240 cumulative equivalent minutes at 43 °C (CEM43).

Before the in vivo mouse experiments, the stability and accuracy of the MR thermometry sequences were determined within chicken filet and a 3 % agarose phantom. The stability of the MR thermometry was measured in a chicken filet and in a mouse. The temperature within the chicken filet without heating was measured for 2 min. The accuracy of the MR thermometry was assessed with a 3 % agarose phantom. The agarose phantom was positioned in boiled water to increase the temperature. The temperature changes were measured with MR thermometry. Within both experiments, a fiber optic temperature probe (T1S-2 M probe, Neoptix, Québec, Canada) was positioned within the phantom or chicken filet as a standard of reference. Within the mouse, a rectal thermometer was used as standard of reference.

### Histopathology

Mice were sacrificed 3 days after HIFU treatment. The tumors were removed and inked for visual orientation after which they were fixated in formalin. The tumors were cut in half, parallel to the surface of the epidermis using the same orientation as the T2W MR imaging made after treatment, under visual inspection. After paraffin embedding, 4-μm thick tissue sections were stained with hematoxylin and eosin (H&E). Immunohistochemical staining by F4/80 was used to investigate macrophage infiltration in the tumor. Paraffin tissue sections (4 μm) were deparaffinized and subjected to antigen retrieval by citrate (10 mM) with microwave treatment for 10 min. Then the tissue sections were incubated with serum to block aspecific bindings, after which the sections were incubated with F4/80 antibodies (1:20 in 2 % normal goat serum (NGS), eBioscience, Vienna, Austria) at room temperature. The first antibody was coupled with a goat anti-rat-biotin antibody (1:100 in 1 % NGS/1 % normal mouse serum) and colored with Fast Red bound to ABC-Horseradish Peroxidase. The sections were counterstained with hematoxylin.

Therapy effects were evaluated and scored on H&E slides by a pathologist. Morphologic changes were evaluated, such as percentage of necrosis, type of tissue damage (apoptosis versus coagulative necrosis), and type of tumor necrosis (confluent versus fractionated). Furthermore, ballooning degeneration, dilatation of capillaries, cysts, and bleeding were recorded. Finally, lymphocytic infiltration, both within and on the tumor edge, was assessed.

## Results

### MR thermometry and ex vivo experiments

The hydrophone measurements of the focal region revealed a cigar shaped volume of 0.5 × 0.5 × 2 mm (full width half maximum (FWHM)). At an acoustic output power of 1.04 W, the peak pressure was 1.8 MPa. The gel pad positioned within the US beam did not show any interference with the US beam or a decrease of the focal pressure, data not shown.

The stability and accuracy of the MR thermometry was evaluated by measuring the temperature within a chicken filet, one mouse and an agarose phantom. The temperature stability was assessed in the absence of heating. The standard deviation of the temperature measured in a chicken filet over 2 min was +/− 0.34 °C with the GRE-flash sequence (temporal resolution 3.8 s) and +/−0.16 °C with the EPI sequence (temporal resolution 1.9 s). The temperature accuracy was determined while heating up an agarose phantom. The average deviation between the MR thermometry and the fiber optic temperature probe was −0.21 °C (±0.52) and 1.6 °C (±1.8), respectively, with the GRE-flash and EPI sequences. Within a non-heated area of the mouse tumor MR thermometry with the EPI sequence revealed a variation of +/−0.31 °C with baseline corrections.

### Mice studies

The mean tumor dimensions were *A* = 8.7 mm (±1.5) and *B* = 7.3 mm (±1.1) on the day of treatment. The mean tumor volume of the mice was 194 mm^3^, with a range between 111 and 285 mm^3^, but one mouse had a relatively small (82 mm^3^) and another a large (364 mm^3^) tumor in contrast to the other tumors. For T-HIFU or M/inT-HIFU treatment, respectively, three or six sonications were positioned within the tumor except one mouse of the M-HIFU group with a tumor size of 82 mm^3^, for which only two focused sonications were applied. One mouse was excluded due to unexpected tumor burst before treatment, leaving a total of 17 mice which were treated with HIFU. Six of them in the thermal HIFU group, 5 in the M-HIFU, and 6 mice in the inT HIFU group. Three mice were used as a control. In three mice, tumor growth was restricted by adjacent muscle tissue, which might have influenced the treatment due to difference in absorption rate of the tissue. The ultrasound beam path was clearly visualized in all mice in T1W MR images (Fig. 2a).

The body temperature during treatment of the anesthetized mice ranged between 34 and 37 °C. Two mice had to be repositioned due to air bubbles within the US beam path, which resulted in a drop of the body temperature (30 °C), but the body temperature was stabilized after repositioning, and both mice recovered normally after treatment. In all experiments the focus of the HIFU system was well aligned after one or two test pulses inside the phantom cube. As observed on the MR images and histopathological slices, all ablations were accurately positioned inside the tumor in all mice. No skin burns were observed immediately after the HIFU treatment. All mice (*n* = 17) retained complete use of the treated leg within 15 min after treatment without showing signals of pain.

Two mice of the T-HIFU group showed a small skin defect covering the tumor 2 and 3 days after treatment. They had some difficulties using the leg with the tumor, possibly due to necrosis of the tumor directly underneath the skin or heating of tissue next to the tumor. Two mice of the inT-HIFU group also revealed small skin defects on top of the tumor. However, they did not show any discomfort and used their legs properly. The size of the tumors of five mice treated with T-HIFU ablation, four tumors of the inT-HIFU-treated mice, and three tumors of the M-HIFU-treated mice diminished in the days after treatment, which was not seen in the control mice.

### MRI measurements

**Fig. 4:**
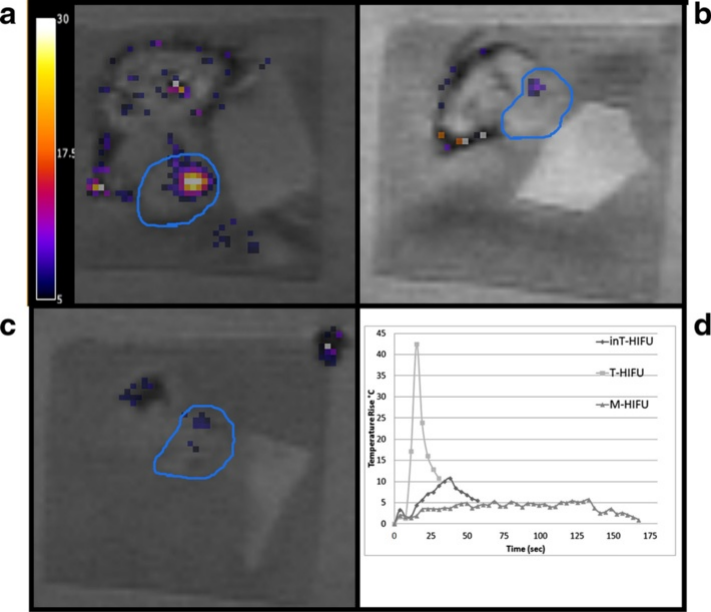
Gradient echo-flash (GRE-FLASH) image with temperature map overlay, during treatment with thermal HIFU (T-HIFU; (**a**)), intermediate HIFU (inT-HIFU; (**b**)), and mechanical HIFU (M-HIFU; (**c**)). Temperature increase of >30, < 13, or <7 °C are reached during treatment (**d**). The tumor is delineated with a *blue line*, the temperature of one focal spot is visualized as a temperature increase

During treatment, the temperature in the tumors was measured in all mice. Each mouse of the T-HIFU group received three focused sonications. In one mouse, the accuracy of the temperature measurements was questionable due to boiling or movement artifacts. The average peak temperature within the other ablation zones (16) was 67.6 °C (±9.6) within 4 s of HIFU ablation (Fig. 4a, d). The mean of the total volume that received at least the lethal dose of 240 CEM43 was 65 mm^3^ (±25). The M-HIFU and inT-HIFU treated mice received 2–6 focused sonications. During inT-HIFU treatment, the temperature slowly increased 9–13 °C within 40 s (Fig. 4b, d). The mechanical HIFU-treated mice presented a small temperature increase of 7 °C within 30 s, after which it stabilized around a peak temperature of maximum 44 °C (Fig. 4c, d). The mice of the inT-HIFU group received a maximum thermal dose of 9.6 CEM43 within each focal spot, while the M-HIFU treated mice showed a thermal dose of less than one CEM43.

**Fig. 5:**
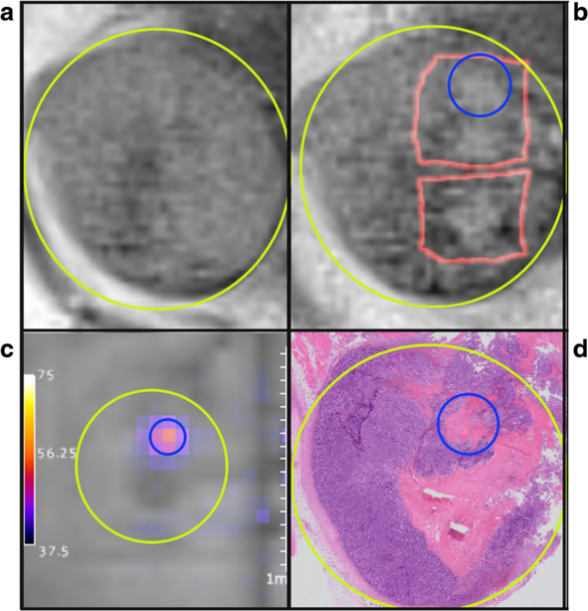
Comparison of the tumor on magnetic resonance (MR) imaging and pathology. T2 weighted (T2W) MR images before (**a**) and immediately after (**b**) 3 ablation spots. *Yellow circle* is the tumor, *red squares* are the thermal dose overlay. **c**:Temperature map (echo planar imaging (EPI) sequence) of one focal spot corresponding with *blue circle* at pathology and T2W image after treatment. **d** hematoxilin and eosin slice of the tumor 3 days after treatment. Showing a focal necrotic region and a confluent necrotic region, caused by heat distribution resulting in fusion of the lesions in the days after treatment

The magnitude MR images recorded during thermal treatment showed a transient large signal drop inside the focal region. The intensity immediately returned after the HIFU pulse was turned off. This was probably the result of local boiling. Because of this, with the real time EPI sequence, the temperature in three focal ablation spots could not be determined accurately. Very little or no signal drop was observed during inT-HIFU or M-HIFU ablation. In the high resolution anatomical T2W MR images directly after treatment, in three out of six T-HIFU treated mice, high intensity focal spots were visible within the tumor after ablation which correlated, by visual orientation, with the lesion found on histopathology sections (Fig. 5). These spots might be the cause of edema due to the treatment. The other three mice also showed lesions on the histopathology, but no clear signal changes were found with T2W imaging. Only, in one mouse subjected to inT-HIFU, the signal intensity in T2W MR images decreased after treatment. Within the M-HIFU-treated group, no signal changes were seen.

### Tumor appearance

**Fig. 6:**
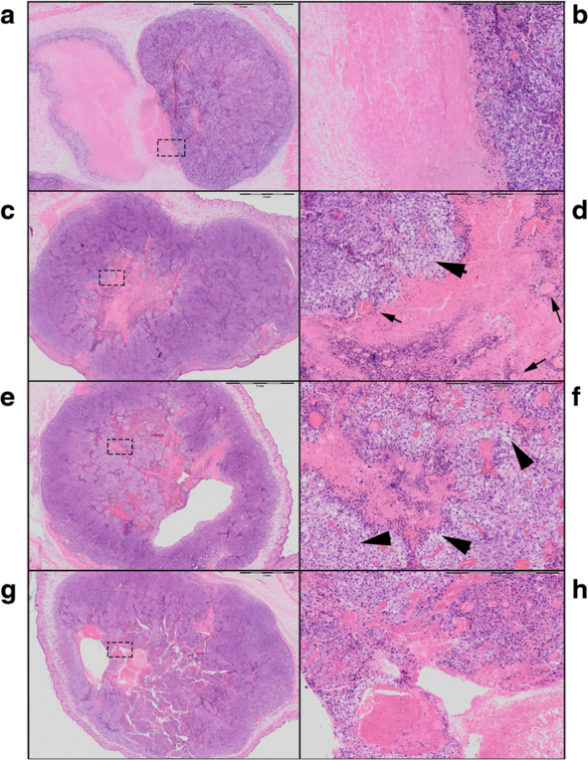
Digitalized hematoxilin and eosin stained sections of the mice tumors. 1× magnification of the tumor control (**a**), thermal (**c**), intermediate (**e**), and mechanical high intensity focused ultrasound treatment (**g**). Magnification of 6.6× of the corresponding rectangle on the left: **a**, **b** well demarcated “old” necrosis without ballooning degeneration surrounding the necrotic area. **c**, **d** Large pink necrotic area in the center of the tumor surrounded with ballooning degeneration (*arrow head*) and dying cells and remnants of vessels (*arrow*) within the necrotic lesion. **e**, **f** Fractionated tissue with islands of necrosis and ballooning degeneration (*arrow heads*) of cells. **g**, **h** Fractionated tissue with bleeding and formation of a cavity filled with erythrocytes

Histologically, the different focal ablations were not recognized as separate spots in the sections from tumors isolated 3 days after HIFU ablation. The H&E staining revealed clear morphologic changes in the tumor tissue, but the necrotic area after thermal ablation appeared as a single big lesion at 3 days after treatment (Fig. 6), probably due to heat diffusion. The T-HIFU group showed large regions of coagulative necrosis (47 % ±11) of the tumor, corresponding with a lesion with an approximate diameter of 4.6 mm (±0.6). The control group also showed necrosis (33 % ±12 over a diameter of 2.8 mm ±0.3), which is normal necrosis of the inner part of larger tumors. However, this was a homogeneously appearing old necrotic area with sharp edges, while T-HIFU-treated tumors had more coarse bordered necrotic tissue, which still contained areas with irreversible dying cells. The necrotic region was surrounded by ballooning degeneration (cell swelling, which is an early stage of cell apoptosis). Five of the six T-HIFU-treated mice also presented vessel remnants within the necrotic regions, which were not seen in the necrotic regions of the control tumors (Fig. 6a–d).

Both the M-HIFU and inT-HIFU group revealed less coagulative necrosis, and more fractionated necrosis and apoptotic cells, compared to one big necrotic region seen in the T-HIFU-treated group. These tumors showed islands of necrosis within vital tumor cell areas. The total necrosis within the treated area was 26 % (sd. 11 %) in the inT-HIFU group and 22 % (sd. 9 %) in the M-HIFU-treated mice. Histopathologically, the H&E sections revealed similar results between the M-HIFU or inT-HIFU ablation settings. In both groups, some hemorrhage and ballooning degeneration was observed. In two mice of the inT-HIFU-treated mice, small pseudocysts within the tumor were found, most of which were filled with erythrocytes (Fig. 6e–h).

All tumors showed moderate lymphocyte infiltration surrounding the tumor. The M-HIFU and inT-HIFU treated mice also showed additional lymphocyte infiltration within the center of the tumor, without differences between the two other groups. In all mice, the F4/80 macrophage staining detected macrophages in the immediate surroundings of the tumor and sometimes near fragmented necrotic regions. However, no quantitative differences were found between the three treatment methods at the time points analyzed.

## Discussion

A new MR-guided HIFU setup for the treatment of small animals was designed and evaluated with three different ablation methods. This setup can be used to distinguish between thermal and mechanical ablation methods. The small focus (0.5 × 0.5 × 2.0 mm FWHM) of the HIFU system results in a high ablation accuracy. The HIFU focus can accurately be positioned within the tumor with minimal to no damage to the surrounding tissue, such as the skin or leg muscles. In combination with the high-field small animal MR system, the temperature within the focal region and surrounding tissue can accurately (+/−0.31 °C) be measured with a high spatial resolution in real time (temporal resolution 1.9 s). Thus, it is now feasible to do larger follow-up studies in mice after MR-guided HIFU treatment to improve treatment methods and monitoring of tumor response.

Two different thermometry methods were used, a GRE-FLASH sequence and an EPI sequence. With the GRE-FLASH sequence, the temperature can only be visualized after each sonication instead of real time visualization due to technical limitations of the sequence. Using the EPI sequence, a thermal map can be displayed immediately during the ablation. The GRE-FLASH sequence created accurate thermometry maps (temperature uncertainty <0.52 °C) and anatomical imaging with a relatively high spatial resolution (0.78 × 0.78 × 1.5 mm^3^, five slices), each 3.8 s. This allowed controllable HIFU and proper follow-up. However, due to the interleaved ablation and visualization of the thermal map, occasionally heating of the surrounding tissue was noticed after ablation, which suggests that direct visualization is required to observe heating of surrounding tissue on time. Furthermore, the fast temperature rise combined with a relatively low temporal resolution (3.8 s) might result in under-sampled and inaccurate temperature measurements due to partial volume effects. With the real time EPI sequence, a less accurate thermometry map was obtained (uncertainty <1.8 °C), in comparison with the GRE-FLASH sequence, and a decreased contrast of the images, making tumor border detection more difficult. However, due to the fast imaging (1.9 s per dynamic per 4 slices), less under-sampling occurred. Also a slice perpendicular to the transversal slices was used to visualize the tissue above the focus of the US beam. Due to the direct visualization of the tumor and surrounded tissue, immediate abortion of the ablation was feasible and any heating of surrounding tissue was noticed immediately. Optimization of the real time imaging is required for better visualization of the tumor margins. While the focal region was easily visualized with T-HIFU or inT-HIFU ablation, during M-HIFU treatment, the temperature change was limited (<7 °C increase) and little or no signal changes were observed in the images during treatment. This results in difficulties to confirm the location of the focus in real time and to determine the treatment size created by M-HIFU treatment. Possibilities to visualize the focal zone with mechanical HIFU treatment might be MR acoustic radiation force imaging [28].

Only in three out of six T-HIFU-treated mice, high intensity focal spots were seen in T2W MR images after treatment, while all mice showed histopathologic changes. This might be because T2W MR images were acquired immediately after treatment. Tissue response might become more apparent some hours after treatment [29]. Next to T2W MR imaging, other MR imaging techniques such as (dynamic) contrast-enhanced MR imaging, diffusion weighted, or T2* weighted MR imaging could be used for treatment evaluation and follow-up [21, 30–33]. It has previously been shown that the permeability of vessels increases, or the vessels are destroyed by mechanical destruction for example due to cavitation effects [8]. The permeability decreases due to tissue necrosis by thermal destruction of tissue, which results in an increase and decrease of contrast uptake [34]. Therefore, contrast MR imaging could visualize both thermal and mechanical HIFU treatment response and to help distinguish the thermal or mechanical bioeffects of different ablation methods.

Although no significant changes were observed in the MR images directly after treatment, microscopic changes due to treatment were found in all tumors. Instead of treating the entire tumor, only a small amount (3–6) of focal ablations was positioned inside the tumor. Histopathologically, the thermal-treated mice revealed more confluent coagulation necrosis, while both M-HIFU- and inT-HIFU-treated groups showed more fractionated tissue necrosis and hemorrhage. These effects are similar as found in earlier studies on sheep, rats, or rabbits [8, 35, 36]. The mechanical effects were limited compared to thermal effects, still some dying cells and living cells were found between the fractionated tissues 3 days after the ablation. Real cavitation effects were not found, possibly because only transient damage was created while the tumor was removed after 3 days. Further, increasing the acoustical output power might be a solution for a more complete mechanical destruction of the tissue, as is shown in boiling histotripsy ablation methods [6, 14]. In future studies, a longer follow-up after treatment is of interest to evaluate further tumor responses after the different ablation techniques.

## Conclusion

In this study, a stable MR guided HIFU treatment setup is designed to treat mice using either T-HIFU, M-HIFU, or an inT-HIFU approach. It is shown that HIFU ablation of murine melanoma tumors is feasible and causes different pathologic effects within the tumor with high accuracy, without damaging the surrounding tissue and with little or no damage to the skin. This design allows to improve and to investigate different HIFU treatment methods and their effects in vivo for larger cohort of animals.
